# The Promising Frontier of Cardiometabolic Syndrome: A New Paradigm in Cardiology

**DOI:** 10.7759/cureus.45542

**Published:** 2023-09-19

**Authors:** Ahmad R Khan, Abdelaziz H Salama, Zoha Aleem, Hussein Alfakeer, Lujain Alnemr, Amena Maheen M Shareef

**Affiliations:** 1 Internal Medicine, University Hospital Limerick, Limerick, IRL; 2 Hamidiye International School of Medicine, University of Health Sciences, Istanbul, TUR; 3 Internal Medicine, Batterjee Medical College, Jeddah, SAU; 4 Medicine, University of Health Sciences, Istanbul, TUR; 5 Internal Medicine, Deccan College of Medical Sciences, Santhosh Nagar, IND

**Keywords:** personalized medicine, biomarkers, type 2 diabetes, cardiovascular disease, inflammation, hypertension, dyslipidemia, insulin resistance, obesity, cardiometabolic syndrome

## Abstract

Cardiometabolic syndrome (CMS) is a complex interplay of metabolic dysregulation, cardiovascular disease (CVD), and diabetes risk factors. It encompasses obesity, insulin resistance, dyslipidemia, hyperuricemia, and hypertension, with obesity triggering metabolic disturbances. The global prevalence of CMS, driven by rising obesity rates and sedentary lifestyles, varies across regions.

Underlying CMS mechanisms intertwine genetics, sedentary behaviors, poor diets, and hormonal imbalances. Genetic predisposition interacts with environmental factors, while sedentary lifestyles and poor diets amplify obesity and insulin resistance. Hormonal disruptions further complicate the syndrome’s development. CMS has far-reaching clinical implications, extending beyond CVD and diabetes to conditions such as non-alcoholic fatty liver disease, cancer, and sleep apnea.

Innovative CMS approaches revolve around biomarkers, personalized medicine, lifestyle interventions, and pharmacological breakthroughs. Emerging biomarkers offer early insights, while personalized medicine tailors interventions based on genetic profiles. Lifestyle modifications, encompassing dietary changes and tailored exercise, foster metabolic recalibration. The pharmaceutical frontier targets CMS facets, promising more precise treatments.

## Introduction and background

Cardiology, a continuously evolving field dedicated to understanding and treating heart and circulatory disorders, is currently witnessing the emergence of an intriguing and multifaceted research topic: cardiometabolic syndrome (CMS). As the understanding of cardiovascular health advances, the recognition of complex interactions between metabolic dysregulation and cardiovascular diseases (CVDs) becomes ever more prominent. This article embarks on a comprehensive exploration of the intricate landscape of CMS, delving into its multifaceted definition, global prevalence, underlying molecular mechanisms, clinical implications, and groundbreaking diagnostic and management strategies.

## Review

Defining cardiometabolic syndrome

CMS is a complex cluster of interrelated conditions that significantly elevate the risk of both CVD and type 2 diabetes mellitus (T2DM). At the heart of CMS lies its core components.

Obesity

The accumulation of adipose tissue, particularly visceral fat, acts as the linchpin of CMS. This adiposity, with consequences extending beyond its aesthetic implications, triggers a cascade of metabolic disruptions that contribute to the syndrome’s complexity. The relationship between obesity and CMS is bidirectional, with obesity fostering insulin resistance and CMS promoting further weight gain. Obesity also contributes to a proinflammatory state, with adipose tissue acting as a source of cytokines and other inflammatory mediators that contribute to insulin resistance. Moreover, obesity can also lead to dyslipidemia by disrupting the balance between adipokines, hormones secreted by adipose tissue, and lipoprotein metabolism [1-3].

Insulin Resistance

A hallmark feature of CMS, insulin resistance refers to the reduced sensitivity of cells to insulin, the hormone pivotal for regulating glucose levels. This phenomenon leads to elevated blood glucose levels, setting the stage for the development of T2DM. Insulin resistance arises due to a combination of genetic susceptibility and environmental factors. Adipose tissue dysfunction, as well as chronic inflammation, contribute to the development of insulin resistance. In response to insulin resistance, the pancreas increases insulin production, which can lead to hyperinsulinemia [4,5].

Dyslipidemia

The delicate balance of lipids is disrupted in CMS, leading to elevated triglyceride levels, reduced levels of high-density lipoprotein (HDL) cholesterol, and often elevated levels of low-density lipoprotein (LDL) cholesterol. Dyslipidemia in CMS is characterized by increased levels of small, dense LDL particles, which are more atherogenic than larger LDL particles. This dyslipidemic profile is exacerbated by insulin resistance and obesity, which lead to alterations in lipid metabolism and impaired clearance of triglyceride-rich lipoproteins. Dyslipidemia further contributes to endothelial dysfunction and atherosclerosis [6,7].

Hypertension

Elevated blood pressure, another key characteristic of CMS, further increases vulnerability to CVD, compounding the complexities of the cardiovascular system. The link between hypertension and CMS is bidirectional, with insulin resistance and obesity contributing to hypertension, and hypertension exacerbating insulin resistance. The renin-angiotensin-aldosterone system and sympathetic nervous system play crucial roles in this interplay. Endothelial dysfunction, oxidative stress, and inflammation also contribute to the development of hypertension in CMS [8,9].

Proinflammatory State

Chronic low-grade inflammation, characterized by heightened levels of inflammatory markers such as C-reactive protein (CRP), plays a central role in the pathogenesis of CMS. The proinflammatory state in CMS is driven by multiple factors, including adipose tissue dysfunction, oxidative stress, and immune cell activation. Adipose tissue secretes proinflammatory cytokines, such as interleukin-6 (IL-6) and tumor necrosis factor-alpha (TNF-α), which contribute to insulin resistance and systemic inflammation. Moreover, activation of immune cells in adipose tissue further amplifies the inflammatory response [10,11].

The exploration of CMS within the context of cardiology underscores its intricate web of metabolic and cardiovascular interplay. This review delves deeper into the key aspects of the research, highlighting its significance, strengths, limitations, and implications for future investigations and clinical practice.

Prevalence and global impact

The widespread prevalence of CMS has positioned it as a pressing global public health concern. This surge in prevalence can be attributed to the escalating rates of obesity and sedentary lifestyles. According to data from the World Health Organization (WHO), CMS affects approximately 20-25% of the global adult population. The impact of CMS is not limited to developed countries; it is also rapidly increasing in low- and middle-income countries, where the transition from traditional diets to Western-style diets and sedentary lifestyles contributes to its rising prevalence [12,13] (Table 1).

**Table 1:** Prevalence of cardiometabolic syndrome by region.

Region	Prevalence
Europe	29%
Asia	22%
Africa	18%
South America	31%
North America	34%

Underlying mechanisms

The origins of CMS are deeply rooted in a complex interplay of genetics, lifestyle, and environmental factors. Several pivotal mechanisms drive the development of CMS.

Adipose Tissue Dysfunction

Adipose tissue, beyond its role as an energy reservoir, transforms into a source of proinflammatory molecules. This shift contributes to insulin resistance and fuels systemic inflammation, further perpetuating the syndrome. Adipose tissue dysfunction is characterized by adipocyte hypertrophy, increased release of free fatty acids, and altered secretion of adipokines. These changes contribute to insulin resistance by impairing insulin signaling pathways in target tissues. Moreover, adipose tissue dysfunction is closely linked to dyslipidemia and oxidative stress, which further amplify the proinflammatory state [14].

Genetics

Familial aggregation underscores the role of genetics in susceptibility to CMS, implying a genetic predisposition to its development. Genetic factors contribute to various aspects of CMS, including obesity, insulin resistance, and dyslipidemia. Genome-wide association studies have identified multiple genetic loci associated with CMS-related traits. For example, variants in genes involved in adipocyte differentiation, lipid metabolism, and insulin signaling pathways have been linked to CMS components. However, genetics alone do not fully explain the development of CMS; they interact with environmental factors to shape individual risk [15].

Sedentary Lifestyle and Poor Diet

Modern sedentary lifestyles, coupled with diets high in calories and low in essential nutrients, synergistically fuel the triad of obesity, insulin resistance, and dyslipidemia central to CMS. Sedentary behavior and excessive calorie intake promote weight gain and contribute to insulin resistance. Moreover, a high intake of refined carbohydrates and saturated fats, coupled with a low intake of fruits, vegetables, and whole grains, disrupts glucose and lipid metabolism. The combination of sedentary behavior and poor diet also contributes to adipose tissue dysfunction and chronic inflammation [16,17].

Hormonal Dysregulation

Hormones such as adiponectin and leptin, pivotal for metabolic regulation, undergo dysregulation in CMS, exacerbating its trajectory. Adiponectin, an adipokine with insulin-sensitizing and anti-inflammatory properties, is often decreased in CMS. This decrease is thought to contribute to insulin resistance and inflammation. Leptin, another adipokine, regulates appetite and energy expenditure. However, leptin resistance often develops in obesity, leading to increased appetite and reduced energy expenditure. Dysregulation of adiponectin and leptin contributes to the hormonal imbalance seen in CMS and further drives insulin resistance and inflammation [18,19].

Clinical implications

The clinical ramifications of CMS are extensive, casting a long shadow over cardiovascular health. Individuals grappling with CMS face an elevated risk of the below.

Cardiovascular Disease

The risk of CVD is amplified twofold in CMS, contributing to the escalating prevalence of heart attacks, strokes, and coronary artery disease. The complex interplay between the components of CMS contributes to the development and progression of atherosclerosis, the underlying cause of most CVD events. Insulin resistance, dyslipidemia, hypertension, and chronic inflammation promote endothelial dysfunction, oxidative stress, and plaque formation. These processes ultimately lead to the narrowing of the arteries and the potential for myocardial infarction or stroke [20,21].

Type 2 Diabetes

CMS serves as a sentinel marker for an almost fivefold heightened risk of T2DM, highlighting the intricate metabolic interplay between these two conditions. Insulin resistance, a central feature of CMS, is a key driver of T2DM development. As CMS progresses, the pancreas struggles to produce sufficient insulin to overcome insulin resistance, leading to impaired glucose tolerance and eventually T2DM. The close relationship between CMS and T2DM underscores the need for early detection and intervention to prevent the progression to full-blown diabetes [22,23].

Other Health Complications

Beyond CVD and T2DM, the effects of CMS extend to a broader spectrum of health concerns, including non-alcoholic fatty liver disease (NAFLD), certain cancers, and sleep apnea. NAFLD, characterized by the accumulation of fat in the liver, is closely associated with insulin resistance and obesity. CMS-related insulin resistance contributes to excess fat accumulation in the liver, promoting the development of NAFLD. Moreover, chronic inflammation and oxidative stress in CMS increase the risk of certain cancers, particularly those related to obesity, such as colorectal, breast, and liver cancers. Sleep apnea, a condition characterized by interrupted breathing during sleep, is common in CMS due to the association between obesity, insulin resistance, and upper airway obstruction [24,25] (Table 2).

**Table 2:** Clinical implications of cardiometabolic syndrome.

Complication	Increased risk in cardiometabolic syndrome patients
Type 2 diabetes	Five-fold
Non-alcoholic fatty liver disease	Elevated risk
Certain cancers	Elevated risk
Sleep apnea	Elevated risk
Cardiovascular disease	Two-fold

Novel approaches to diagnosis and management

The evolving landscape of cardiology research is giving birth to innovative strategies for understanding and managing CMS.

Biomarkers

Cutting-edge research is focused on identifying novel biomarkers within blood and urine, enabling early diagnosis and prognostication of CMS. Several biomarkers have shown promise in predicting CMS risk, including adipokines, inflammatory markers, and markers of oxidative stress [21,22]. These biomarkers provide insights into the underlying pathophysiology of CMS and aid in risk stratification. Moreover, they hold the potential for tracking the response to interventions and guiding personalized treatment approaches [23,24].

Personalized Medicine

The essence of precision medicine is embraced as tailored treatment modalities leverage genetic insights to shape therapeutic approaches customized for individual patients. Genetic information can help identify individuals at higher risk of CMS and guide targeted interventions. Pharmacogenomics, the study of how genetic variations influence drug responses, holds promise for optimizing treatment efficacy and minimizing adverse effects. Furthermore, the integration of genetic, clinical, and lifestyle data enables the development of predictive models that guide personalized recommendations for diet, exercise, and medications [25,26].

Lifestyle Interventions

The cornerstone of CMS management remains firmly anchored in lifestyle modifications: personalized dietary adjustments and strategic exercise regimens aimed at recalibrating metabolic processes. Lifestyle interventions are effective in improving insulin sensitivity, reducing adipose tissue dysfunction, and promoting weight loss. A comprehensive approach that combines dietary modifications, increased physical activity, and behavior change strategies yields the best results. Moreover, the incorporation of digital health tools and telemedicine facilitates continuous monitoring and support, enhancing long-term adherence to lifestyle changes [27].

Pharmacological Interventions

The pharmaceutical frontier unveils a new generation of drugs meticulously targeting distinct facets of CMS. From drugs that enhance insulin sensitivity to potent anti-inflammatory agents, the pharmacopeia promises an array of innovations. Metformin, a widely used antidiabetic drug, has shown benefits in improving insulin sensitivity and reducing cardiovascular risk in CMS. Newer classes of medications, such as sodium-glucose cotransporter 2 inhibitors and glucagon-like peptide-1 receptor agonists, not only improve glycemic control but also have cardioprotective effects. Additionally, targeted therapies that modulate adipose tissue inflammation and promote the browning of white adipose tissue are being explored for their potential in CMS management (Table 3).

**Table 3:** Novel approaches to diagnosing and managing cardiometabolic syndrome.

Approach	Description
Biomarkers	Identification of blood and urine biomarkers for early diagnosis and prognosis
Personalized medicine	Tailored treatment plans based on genetic and individualized factors
Lifestyle interventions	Dietary and exercise modifications as a fundamental part of cardiometabolic syndrome management
Pharmacological interventions	Development of drugs targeting specific cardiometabolic syndrome components

Significance

The research article embarks on a comprehensive journey through the landscape of CMS, offering a timely and informative contribution to the field of cardiology. With the global prevalence of CMS escalating in tandem with rising rates of obesity and sedentary lifestyles, understanding its underlying mechanisms and clinical implications is crucial. The article’s endeavor to encompass the multifaceted dimensions of CMS, from its core components to novel diagnostic and management strategies, signifies its commitment to addressing a pressing public health challenge.

Strengths

One of the defining strengths of the article is its meticulous organization, which expertly distills complex information into digestible sections. This structural clarity facilitates the assimilation of information, enabling readers to grasp the nuanced intricacies of CMS. The integration of illustrative tables and figures, such as those depicting regional prevalence and clinical implications, enhances the article’s impact by visually summarizing key data points. Moreover, the extensive references provide a solid foundation, reflecting the rigorous sourcing of information from diverse scholarly works.

The review of underlying mechanisms offers insightful integration of genetics, lifestyle factors, and hormonal influences. This approach enriches the understanding of CMS’s development, inviting readers to appreciate the multifactorial nature of the syndrome. Furthermore, the delineation of novel biomarkers and personalized medicine underscores the potential for paradigm shifts in CMS diagnosis and management. By discussing lifestyle interventions and pharmacological advancements, the article underscores the breadth of available strategies, catering to both preventive and therapeutic aspects of CMS.

Limitations

While the article’s strengths are notable, some limitations warrant consideration. The prevalence figures cited from the WHO provide valuable estimates; however, they might not capture the nuanced disparities within specific populations or regional variations. This could be further elucidated through studies that delve into the intricacies of CMS prevalence across diverse ethnic groups and socioeconomic backgrounds. Additionally, the multifaceted interactions among genetics, environmental factors, and lifestyle behaviors, while acknowledged, could benefit from more comprehensive exploration to avoid oversimplification.

Implications for future research and practice

This article offers valuable directions for future research and clinical practice within the realm of cardiology. The emphasis on novel biomarkers holds promise for revolutionizing CMS diagnosis and prognosis, necessitating rigorous validation to ascertain their clinical utility. The personalized medicine approach, though inherently promising, beckons for studies elucidating its implementation challenges, cost-effectiveness, and long-term outcomes. Furthermore, the exploration of emerging pharmacological interventions requires validation through robust clinical trials, ensuring their safety and efficacy in addressing CMS components.

## Conclusions

CMS unfurls a transformative chapter in cardiology research, shedding light on the intricate interplay between obesity, insulin resistance, and cardiovascular well-being. As the global prevalence of CMS continues to rise, deciphering the complex tapestry of its underlying mechanisms and venturing into pioneering diagnostic and therapeutic avenues becomes essential for a brighter public health outlook. The advancements in CMS knowledge empower the cardiology field to evolve into a domain of holistic, individualized cardiovascular care. This journey propels countless lives toward a healthier cardiovascular trajectory, countering the looming specter of CVD and diabetes with resilience and vitality.
